# The Prevalence, Characteristics and Risk Factors of Persistent Symptoms in Non-Hospitalized and Hospitalized Children with SARS-CoV-2 Infection Followed-Up for up to 12 Months: A Prospective, Cohort Study in Rome, Italy

**DOI:** 10.3390/jcm11226772

**Published:** 2022-11-16

**Authors:** Danilo Buonsenso, Ekaterina Pazukhina, Carolina Gentili, Luigi Vetrugno, Rosa Morello, Margherita Zona, Alessia De Matteis, Federico D’Ilario, Roberta Lanni, Teresa Rongai, Patrizia del Balzo, Maria Teresa Fonte, Michele Valente, Cristina De Rose, Daniel Munblit, Louise Sigfrid, Piero Valentini

**Affiliations:** 1 Department of Woman and Child Health and Public Health, Fondazione Policlinico Universitario A. Gemelli IRCCS, Largo A. Gemelli 8, 00168 Roma, Italy; 2 Global Health Research Institute, Istituto di Igiene, Università Cattolica del Sacro Cuore, 00168 Roma, Italy; 3 Dipartimento di Scienze Biotecnologiche di Base, Cliniche Intensivologiche e, Perioperatorie–Sezione di Microbiologia, Università Cattolica del S. Cuore, 00168 Rome, Italy; 4 Laboratory of Health Economics, Institute of Applied Economic Studies, The Russian Presidential Academy of National Economy and Public Administration, 127006 Moscow, Russia; 5 Center for Advanced Financial Planning, Macroeconomic Analysis and Financial Statistics, Financial Research Institute of the Ministry of Finance of the Russian Federation, 127006 Moscow, Russia; 6 Department of Medical, Oral and Biotechnological Sciences, University of Chieti-Pescara, 66100 Chieti, Italy; 7 Pediatra di Libera Scelta, Federazione Italiana Medici Pediatri (FIMP), 00168 Rome, Italy; 8 Department of Paediatrics and Paediatric Infectious Diseases, Institute of Child’s Health, Sechenov First Moscow State Medical University (Sechenov University), 123337 Moscow, Russia; 9 Inflammation, Repair and Development Section, National Heart and Lung Institute, Faculty of Medicine, Imperial College London, London W2 1PG, UK; 10 Research and Clinical Center for Neuropsychiatry, 115419 Moscow, Russia; 11 International Severe Acute and emerging Infection Consortium (ISARIC), Global Research Collaboration for Infectious Disease Preparedness, Pandemic Sciences Institute, Nuffield Department of Medicine, University of Oxford, Oxford OX3 7LG, UK

**Keywords:** Long COVID, post-COVID condition, children, SARS-CoV-2, COVID-19

## Abstract

Previous studies assessing the prevalence of COVID-19 sequelae in children have included either a small number of children or a short follow-up period, or have only focused on hospitalized children. We investigated the prevalence of persistent symptoms amongst children and assessed the risk factors, including the impact of variants. A prospective cohort study included children (≤18 years old) with PCR-confirmed SARS-CoV-2 infection. The participants were assessed via telephone and face-to-face visits at 1–5, 6–9 and 12 or more months post-SARS-CoV-2 diagnosis using the ISARIC COVID-19 follow-up survey. Of the 679 children enrolled, 51% were female; 488 were infected during the wild virus wave, and 29 were infected with the Alpha, 42 with the Delta and 120 with the Omicron variants. Fatigue (19%), headache (12%), insomnia (7.5%), muscle pain (6.9%) and confusion with concentration issues (6.8%) were the most common persistent symptoms. Families reported an overall improvement over time, with 0.7% of parents interviewed at 12 months or more of the follow-up period reporting a poor recovery. Patients that had not recovered by 6–9 months had a lower probability of recovering during the next follow-up period. Children infected with a variant or the wild virus had an overall similar rate of persistent symptoms (although the pattern of reported symptoms differed significantly) and recovery rates. Conclusions: Recovery rates after SARS-CoV-2 infection improved as time passed from the initial infection, ranging from 4% of children having poor recovery at 1–5 months’ follow-up to 1.3% at 6–9 months and 0.7% at 12 months. The patterns of persistence changed according to the variants involved at the time of infection. This study reinforces that a subgroup of children develop long-lasting persistent symptoms and highlights the need for further studies investigating the reasons behind the development of PCC.

## 1. Introduction

More than two years after the beginning of the COVID-19 pandemic, there is increasing evidence that some people do not fully recover after an acute SARS-CoV-2 infection. It is now clear that a proportion of people develop Long COVID or the Post-COVID Condition (PCC), which can have severe impacts on daily activities and the quality of life [1]. Thus far, many studies from different regions have documented the burden of PCC in adults for up to one year of follow-up, characterizing the main symptoms and sequelae, identifying the risk factors and exploring the possible pathophysiology [2]. However, the evidence of PCC in children is still limited and controversial. A lack of a pediatric research and clinical case definition and of research investment is adding to the challenge. Initial studies have shown the relatively high rates of children presenting with persistent symptoms, ranging from 2 to 40% [3]. However, many were based on self-completed, uncontrolled online surveys or interviews during the initial pandemic waves, when full or partial lockdowns were still in place, which may have contributed to an overestimation of the PCC risk in children. Subsequently, more rigorous observational studies using standardized protocols and tools identified more conservative prevalence estimates of 1 to 2%, with many children recovering after six to nine months [3]. Considering that it is hard to ascertain and identify children who have never had a SARS-CoV-2 infection, estimating the real burden of PCC in children is becoming more difficult. It is clear from the available data from researchers, pediatricians, children and young people affected, that the Post-COVID Condition (PCC), although affecting children at a lower rate than adults, can have a severe impact on the children affected [4,5]. PCC in children can impact their ability to engage in education and physical and social activities, which may have a long-standing impact on their future [3].

With the growing clinical experience in the field, it is becoming clearer as to what PCC may encompass. Specifically, a recent Delphi process developed by international experts and families led to a definition of PCC as being characterized by persistent symptoms which were: lasting for at least 12 weeks, not present before the COVID-19 infection, unexplained by other known conditions and having a negative impact on daily life [6]. This definition highlights the importance of trained professionals interviewing the children and parents, because a self-completed survey may misconstrue the mild symptoms, not affecting daily life or complained of before the COVID-19 infection, as symptoms of PCC. A similar approach has been used to prospectively investigate the burden of PCC in a relatively large cohort of hospitalized children in Russia up 12 months after initial infection, showing that PCC improves over time, but persists in about 10% of children one year after COVID-19 infection [4]. However, since the large majority of children develop a mild disease not requiring hospitalization, it is important to understand the long-term outcomes of this cohort.

For this reason, we performed this longitudinal observational study of a large cohort of non-hospitalized and hospitalized children with proven SARS-CoV-2 infections to investigate the characteristics of persistent symptoms for up to 12 months after the initial infection and according to the main circulating variants at the time of the initial infection.

## 2. Materials and Methods

This is a prospective longitudinal cohort study including children diagnosed with SARS-CoV-2. The methods have been described in previous papers [4,5]. The relevant institution is a regional COVID-19 referral center for adults and has a dedicated Pediatric Infectious Disease In-patient Unit and an outpatient Pediatric Post-COVID Unit where the ISARIC survey is used as a screening tool for persistent symptoms. Therefore, both the children hospitalized and the community patients assessed in the outpatient unit were included.

The children (≤18 years old) diagnosed with SARS-CoV-2 infection using the reverse transcriptase polymerase chain reaction (RT-PCR) between 1 April 2020 and 31 April 2022 at the Department of Women and Child Health of the Fondazione Policlinico Universitario A. Gemelli IRCCS of Rome, Italy, were invited to participate. The parents of the children (household index case) were contacted by the pediatric residents between 30 September 2020 and 31 April 2022. The follow-up assessments were conducted via an interview with the parents/caregivers, which was via a phone call, a survey or during a face-to-face outpatient assessment.

The first assessment was made at 1–5 months, followed by a second assessment at 6–9 months of the post-SARS-CoV-2 diagnosis of the index case, and a third assessment at 12 months post-diagnosis. Non-responders were contacted by telephone three times before being excluded.

We used the ISARIC Global COVID-19 follow-up protocol for children and the associated standardized data collection forms. The assessment methodology has been presented in earlier linked studies [4,5,7,8]. Information about the participants’ current health status was assessed using the ISARIC COVID-19 Health and Wellbeing Follow-Up Survey for Children (version 1.0 translated into Italian). The survey assesses the physical and psychosocial health and wellbeing and its impact on daily functioning, behavior, relationships and daily living (available from the coordinator at the available contact, see [6]). The survey documents the data on demographics, pre-existing comorbidities, acute severity and information on the acute phase of the disease (symptoms, comorbidities and clinical outcomes) and its severity (hospital admission, pediatric intensive care (PICU/ICU) and oxygenation). Moreover, data on the parental perception of the changes in their child’s emotional and behavioral status, including the reasons for the observed changes (the direct or indirect impacts of COVID-19 or both), persistent symptoms at the follow-up assessment, the overall health condition compared to prior to the index case SARS-CoV-2 diagnosis and mortality.

Considering that there is not yet a universally agreed definition of the post-COVID condition in children, we decided to describe all the symptoms lasting more than 1 month in children and provide a specific analysis of symptoms persisting >6 months post- SARS-CoV-2 infection, as previous studies have suggested that most children tend to recover within six months from the initial infection [4,5,8].

### 2.1. Inclusion Criteria

Children aged 0–18 years:

- The child sought/needed primary or secondary medical care for COVID-19;
- A laboratory (RT-PCR, COVID-19 antigen tests or SARS-CoV-2 antibody testing) confirmed SARS-CoV-2 infection;
- A period of at least 30 days from the diagnosis of the COVID-19 infection;
- The parent’s/caregiver’s/guardian’s consent to participate.

Patients > 18 years old or with severe neurocognitive disability were excluded, because this would not have allowed a proper assessment of signs and symptoms included in the survey. In addition, we excluded children that were vaccinated before the SARS-CoV-2 infection, because addressing the role of vaccination in preventing PCC was outside the scope of this study, and the time frame of this study would not have allowed the role of vaccination in reducing PCC in children younger than 12 years of age to be addressed, as this vaccination was only available at the end of December. Last, we excluded children that had a SARS-CoV-2 re-infection, because this would have created confusion with the interpretation of new symptoms and in the understanding of which infection would have led to the persistent symptoms.

### 2.2. Outcomes

The primary aim of this study is to characterize the cluster of persistent symptoms in a cohort of children post-SARS-CoV-2 infection in Rome, Italy. The secondary objectives are:

- To characterize the features of persistent symptoms according to the main circulating variants in Italy at the time of the initial SARS-CoV-2 infection;
- To characterize the behavioral and neurocognitive changes after SARS-CoV-2 infection.

### 2.3. Exposure and Outcome Variables

For the purpose of this study, we defined “persistent symptoms” as symptoms that were present at the time of the follow-up interview and had been present for at least 4 weeks since the diagnosis. These were sub-categorized into respiratory, neurological, sensory, sleep, gastrointestinal, general (including headache, malaise, and fatigue), dermatological, cardiovascular, urogenital and musculoskeletal, as informed by the previously published literature and the ISARIC Global Paediatric COVID-19 follow-up working group consensus discussions [8]. Health status was assessed using the EuroQol overall health status tool [8], where zero is categorized as the worst possible health and 100 the best possible health.

In order to define the impact of the main circulating variants at time of infection and the pattern of persistent symptoms, we collected information on the main variants in Italy from the following source and classified the periods of the main SARS-CoV-2 variants in Italy, as follows: before 22 February 2021—wild; between 22 February 2021 and 28 June 2021—Alpha; between 28 June 2021 and 27 December 2021—Delta; and after 27 December 2021—Omicron [9].

### 2.4. Statistical Analyses

Descriptive statistics were calculated for the baseline characteristics. The continuous variables were summarized as median (with interquartile range) and the categorical variables as frequency (percentage). Wilcoxon rank sum test, Fisher’s exact test and Pearson’s Chi-squared test were used to check the difference in proportions between different age groups, sexes and periods. Two-sided *p*-values were reported for all statistical tests; a p-value below 0.05 was considered to be statistically significant. A correlation matrix was used to find associations between the most prevalent persistent symptoms.

We included all participants for whom the variables of interest were available for the final analysis, without including the missing data. The differing denominators used indicate the missing data.

The statistical analysis was performed using the R version 4.0.2. The packages used included dplyr, ggplot2, readxl, gtsummary, skimr, data.table, stringr, janitor, zoo, corrplot, reshape2, tibble, flextable, imputeTS and DescTools.

### 2.5. Ethical Approval

This study was approved by the Ethics Committee of the Fondazione Policlinico Universitario A. Gemelli IRCCS of Rome, Italy (ID3777). Written informed consent was obtained by each parent/carer/guardian for their children’s participation, and from the child as well when older than 12 years of age. Consent was obtained either during an outpatient visit or by contacting the families via a phone call or an e-mail; the study procedures were explained, followed by a consent form emailed to the participants, and returned to the study’s staff.

### 2.6. Patient and Public Involvement

The survey was developed by the ISARIC Global Paediatric and Adult COVID-19 follow-up working groups and informed by a wide range of global stakeholders with expertise in infectious diseases, critical care, pediatrics, epidemiology, allergy-immunology, respiratory medicine, psychiatry, psychology and methodology, and by patient–public representatives including people living with Long COVID and members of the Long COVID Support group. The data collection forms were distributed to the members of the patient groups and suggestions from the parents/caregivers were implemented to ensure the key symptoms and sequelae were assessed.

## 3. Results

### 3.1. Study Population

Of the 679 children enrolled in the study, 51% were female (344) and 49% were male (330). The children below 10 years old numbered 317, with 50% female (157) and 50% male (158); the children 10 years and older numbered 359, with 52%female (186) and 48% male (170). Eighteen (2.6%) children were hospitalized during acute infection and five (0.7%) required admission in the Pediatric Intensive Care Unit. Four hundred and eighty-eight children were infected during the wild virus waves. Children with the Alpha variant numbered 29 and there were 42 with the Delta and 120 with the Omicron variants.

The follow-up was extended through three main periods: 1–5 months, which involved 157 children, 6–9 months, which involved 157 children, and 12 months and more, which involved 154 children. Of the 679 children’s parents, 279 were interviewed only once, 153 were interviewed twice and 30 were interviewed three times.

Details of demographic data of the study population according to the time of follow-up and the comorbidities are presented in Table 1.

### 3.2. Persistent Symptoms

The persistent symptoms reported at different follow-up times from acute infection are reported in Table 2, including analyses by sex and age groups, while the number of persistent symptoms according to the same variables are reported in the Supplementary Table S1. Overall, fatigue, nasal congestion and headache were the most commonly reported symptoms at each time point, although these improved over time. Comparing children by sex, the girls suffered mainly from headache (15%), insomnia (10%) and alternated taste (3.2%). On the other hand, the boys suffered mainly from persistent cough (7%), persistent muscle pain (7.6%), confusion (7.6%), poor appetite (7.3%) and diarrhea (6.4%).

Comparing children by age groups, children under 10 years were more affected by nasal congestion and rhinorrhea (20%), constipation (6.9%) and persistent cough (6.6%). Children 10 years and older were more affected by fatigue (25%), headache (18%), insomnia (11%), persistent muscle pain (9.2%), confusion and lack of concentration (8.7%), joint pain and swelling (6.4%), diarrhea (5.6%), chest pain (5.8%), weight loss (5%), feeling nauseous (4.5%), alternated sense of smell (4.5%), alternated taste (4.5%), pain in breathing (3.8%), hypersomnia (3.1%) and loss of taste (2.8%). The burden of persistent symptoms according to age and sex is reported in the Supplementary Figures S1 and S2.

**Figure 1:**
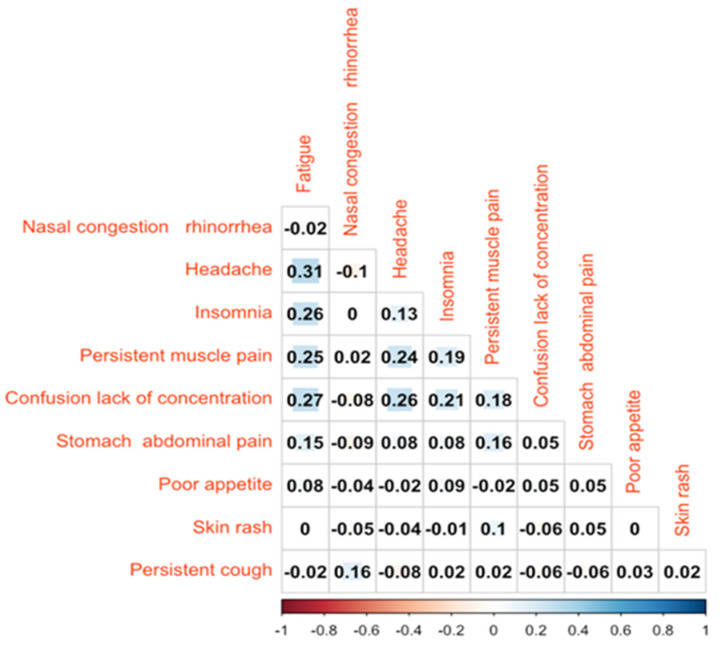
The coexistence of the ten most commonly reported symptoms.

Figure 1 shows the coexistence of at least two symptoms focusing on the ten most common symptoms. Headache and fatigue were the two most-associated symptoms. Fatigue was also reported with insomnia, persistent muscle pain and lack of concentration, but rarely with stomach and abdominal pain. Lack of concentration was associated with fatigue, as mentioned before, headache, insomnia and persistent muscle pain.

We further analyzed the characteristics of persistent symptoms according to the main circulating variants in Italy at the time of initial infection of each patient, and compared children infected during Omicron (n = 120) with those with the wild type (n = 201), at 1–5 months’ follow-up. The main persistent symptoms are reported in Table 3, showing that all subsets of persistent symptoms were more common with the wild variants.

### 3.3. Recovery Rates over Time

Parents interviewed were asked to report the recovery rate of their children on a sliding scale of 1 (worst) to 10 (full recovery). Overall, 86% of parents reported a full or almost complete recovery (8–10/10 score), 11% reported a partial recovery (5–7/10) and 2.6% reported a poor recovery (1–4/10), while from a sub-analysis according to the timing of follow-up, 0.7% of parents reported a poor recovery at 12 months or more of the follow-up (1–4/10). From a sub-analysis by sex and age, boys and the children aged 10 years or more had a higher probability of reporting a poor recovery. Further details are reported in Table 4 and Table S2.

Table 5 shows the dynamics of recovery in the subgroup of patients that underwent repeated follow-ups during different time points. Comparing the recovery rates of those interviewed both at the 1–5 and 6–9 months’ follow-ups, we found that all children reported recovery when reassessed at the 6–9 months’ follow-up (*p* > 0.001). Conversely, the recovery rates remained similar in the group of children interviewed at 1–5 and 6–9 months and those interviewed at 6–9 and >12 months (*p* > 0.05). Recovery rates at the 1-5 months’ follow-up in patients infected with Omicron or the wild type were similar overall (Table S3).

### 3.4. Neurocognitive Impact, Mental Health and Behavioral Changes

Table S4 shows the distribution of patient subgroups across cognitive characteristics by follow-up period, sex and age. Tiredness was the most commonly reported symptom between both females and males at each follow-up, while other issues, including memory and concentration problems, were reported by 1 to 5% of patients according to the specific task analyzed or the timing of the follow-up.

Supplementary Table S5 reports changes in habits, including eating, spending time with friends and school attendance according to the time of follow-up and the sex. In our cohort of female patients affected by Long COVID, 40.9% reported less interest in physical activity at 1–5 months’ follow-up, 25.7% reported less interactions with peers, 29.6% reported less time spent outside, 14.5% reported less school attendance, 22.7% reported changes in their emotions and 12.7% reported modifications in their behavior. The prevalence of all these symptoms is lower at the 6–9 months’ follow-up and at the 12 months’ follow-up, even though 22.5% of females still reported a reduced level of physical activity, 26.7% reported reduced interactions with peers, 17.1% spent less time outside and 12.0% did not attend school. Similar findings, including an improvement over time, were reported by boys.

## 4. Discussion

In this large observational study of 679 children with mostly mild COVID-19, we found that the persistence of symptoms after infection was relatively common at different follow-up periods independent of sex and age. However, the overall feelings of recovery improved as time passed from the initial infection and only 0.7% of families reported a poor recovery at the 12 months’ follow-up.

In further detail, in agreement with several other adult and pediatric studies [3], fatigue, headache, musculoskeletal and gastrointestinal symptoms as well as neurocognitive problems were the most frequently reported in our large pediatric cohort. Interestingly, fatigue, headache and neurocognitive symptoms coexisted more frequently, suggesting that this might be the commonest phenotype of pediatric Long COVID. This subset of symptoms is similar to other CFS/ME reported historically by patients following other viral infections, suggesting that Long COVID may be a form of CFS/ME following SARS-CoV-2 infection [3]. Although several researchers are investigating the origin of these systemic symptoms in Long COVID, so far no proven theories are available.

Conversely, it is easier to understand the gastro-intestinal symptoms like diarrhea, vomiting, nausea, stomach and abdominal pain, which were particularly common at all different timepoints, especially in males and children over 10 years old. This data was not unexpected, given the growing understanding on the role of the GI tract as a site of latency for the virus, or at least parts of it [1,10,11,12]. Several reports have reported that SARS-CoV-2 can be detected in stools for months after the acute phase of the illness, even when the virus cannot be detected in the respiratory tract anymore. Recent data suggest that viral persistence in the gut can alter the local microbiota and stimulate mucosal inflammation, which can explain the GI symptoms and also the other systemic issues complained of by the patients [1]. Importantly, alterations in intestinal permeability have also been linked to the development of MIS-C [13].

On the other hand, the prevalence of symptoms like chest pain, difficulty in breathing, chest tightness, pain in breathing and alternated taste and sense of smell were quite relevant at the beginning of the follow-up, but significantly improved at the 12 months’ follow-up. This suggest that the most severe spectrum of Long COVID with abnormal lung perfusion and chronic endotheliopathy may be rare [14,15,16].

Studies with adults suggest that the female sex is significantly more associated with Long COVID, while in children the reports change according to the available studies [3,4,5]. In a preliminary smaller study, we did not find significant differences in pediatric Long COVID according to sex [8,17,18,19,20], and neither were these the results of this larger report; apart from headache, insomnia, anxiety and depression, which were more frequently noticed in females, all the other sequels are much more common in the male patients, especially the symptoms related to the gastro-intestinal system. These sex-specific clinical phenotypes of Long COVID require further investigation.

According to previous studies [3,8,17,18,19,20], children older than 10 years were more prone to develop sequelae. One explanation could relate to the age-related variations in the expression of the ACE-2 receptors and the TMPRSS2 expression profile at the epithelial sites of the lung and the skin and alterations of the expression of genes in the PBMCs and T cells [21], but also the different contributions of immunological mechanisms that are related to the disease becoming more severe with increasing of age [22,23]. It is also possible that older children have suffered more from the social restrictions and psychological impact of the pandemic, contributing to the reporting of more subtle symptoms [20]. On the other hand, some symptoms may be more difficult to detect in younger children (e.g., fatigue), and this may also contribute to the different burden of the disease in different age groups.

As with adults, in children there are also some comorbidities associated with a higher risk of developing Long COVID: comorbid allergic diseases (eczema, respiratory diseases, allergic rhinitis, food allergy and asthma), neurological diseases, anxiety, excessive weight and heart diseases. The correlation between allergies and pediatric Long COVID was also found in a Russian cohort [8]. Obesity is highly related to both a more severe course of the acute phase and a higher risk of developing Long COVID, partly due to a systemic inflammatory state, proven by a higher level of pro-inflammatory cytokines, including IL-6 and TNF-a, and partly because of the endothelial dysfunction and the increased expression of ACE2 associated with being overweight [24,25]. There is limited information on the association between allergic illnesses and COVID-19 and its sequels. One of the main theories behind allergies is an imbalance between the Th1 and Th2 lymphocytes, where Th2 becomes predominant with a reduction of the Th1 lymphocytes, which represent our first line of defense against external pathogens. When the Th2 lymphocytes become predominant, they can cause an overreaction resulting in allergies or other autoimmune diseases [26,27]. However, it is important to remember that allergic diseases are common and are found with an increasing prevalence in children [27]; this partially explains why the allergic patients’ samples are so varied. About immunological responses, preliminary reports of imbalanced immune responses in children with Long COVID have noted a wide variation of immune phenotypes and an overlap between children who have recovered from Long COVID [28].

Interestingly, persistent symptoms were reported with each one of the different SARS-CoV-2 variants registered in Italy so far. In our study, we have been able to compare the persistent symptoms at 1–5 months’ follow-up in children infected by the wild type or Omicron variants only, because the other variants (Alpha and Delta) were distributed in small numbers during different timepoints not allowing enough data for the comparisons. Interestingly, while the recovery rates were similar in children infected with the Omicron or wild virus, the pattern of persistent symptoms was different, with a significantly lower rate in children infected with Omicron complaining of fatigue, musculoskeletal symptoms and an altered sense of smell. To our knowledge, no previous studies addressed the changes in symptoms according to the infecting variant.

It is important to note that the reported symptoms do not automatically mean a diagnosis of Long COVID, because according to the latest definition the diagnosis of Long COVID requires the exclusion of other comorbidities and a negative impact on daily life. In fact, when families were requested to provide a recovery rate after initial infection, we found that the children with a poor recovery from infection were progressively fewer, from 4% at 1-5 months’ follow-up to 1.3% at 6–9 months and 0.4% at 12 months, in agreement with previous studies that most, but not all, children recover spontaneously [3,8,17,18,19,20]. Interestingly, we found that there was a significant decline in “poor recovery” from the 1 to 5 months’ follow-up, while those with poor recovery at 6–9 months had a good probability of not recovering at 12 months. This finding may have implications, suggesting that a close follow-up may be enough for the first 5 months after infection, while investigations may be reserved for those with persistent symptoms at 6 months after infection.

We also investigated wellbeing and other behavioral changes of this cohort. Overall, the families reported a higher level of tiredness, lack of energy, lack of physical activity, (43.6% of male patients and 40.9% at the 1–5 months’ follow-up and 22.5% of female patients and 18.8% of patients at the 12 months’ follow-up), spending more time at home, which in turn could lead to isolation, depression, anxiety, a growing sense of fear and anger. Also, children in our cohort, both for study and recreational reasons, spent more time on the computer rather than outside with friends. With reference to this, 10.1% of children at the 1–5 months’ follow-up and 5.3% of children at the 12 months’ follow-up reported that they were willing to interact with peers remotely rather than in person. This is in line with other studies that tracked physical activity during COVID-19, such as research conducted in Germany [29]. These symptoms may have been linked to the social restrictions and psychological impact of the pandemic, rather than the virus itself, as also reported by other studies. The pandemic itself has had a significant impact on the psychosocial well-being of children; school closures, social distancing and home quarantine have caused a prolonged period of isolation. Children could not physically be with peers and build friendships, which is fundamental during the developmental ages. These conditions led to an increase in the number of mental health problems in children and adolescents, which is supported by studies showing that loneliness seems to be the main determinant for future mental health problems up to nine years subsequently [20,30].

This study is not without limitations. First, the study is based on self-reported symptoms and mainly on parents reported symptoms. This is because children, especially toddlers, are not able to describe their health conditions properly, it is difficult for them to compare their pre and post-COVID-19 conditions and, as frequently happens in pediatric clinical practice, parents are the best option to gain information about clinical history, and the signs and symptoms and impacts on daily activities. Secondly, this study did not include further clinical, laboratory and radiological investigations for all children interviewed, due to the pandemic conditions and resource limitations. Such further investigations are fundamental for children with multiple symptoms, especially if these symptoms last for more than three months. However, as all the interviews were conducted by trained doctors, we focused on the persistence of symptoms that had not been described before contracting COVID-19. Third, not all children were interviewed at the same time points. Fourth, as we excluded the vaccinated or re-infected children, we could not establish the impact of these two variables on changing the probability of developing persistent symptoms after COVID-19. Last, the rates of persistent symptoms and the recovery rates described in this paper may not strictly reflect the real probability of developing Long COVID in children, because the number of recognized pediatric infections is expected to be underestimated as the majority of children develop asymptomatic or mild infections and are not diagnosed, and therefore the real burden on the children that did not recover at the one year follow-up is probably much lower. Also, we did not include a control group of children that had never had COVID-19. However, it is important to highlight that it is challenging to find an appropriate control group, as most children have had COVID-19 or have been vaccinated. In addition, not all children develop IgG after natural infection or the antibody titer might decay over time, alternatively, the anti N-IgG has lower sensitivity and may not perfectly work in children that have been vaccinated previously.

## 5. Conclusions

In conclusion, in this study which enrolled a large number of children who were followed-up for up to one year, we found that the recovery rates improved as time passed from initial infection. These ranged from 4% of children having poor recovery at the 1–5 months’ follow-up to 1.3% at 6–9 months and 0.7% at 12 months, although if translated into the larger overall numbers of infected children, these may indicate a significant long-term impact of Long COVID in children. The patterns of persistence changed according to the involved variants at the time of infection. This study reinforced that a subgroup of children developed long-lasting persistent symptoms and highlights the need for further studies investigating the reasons behind the development of PCC.

## Figures and Tables

**Table 1:** Distribution of the cohort by period, by sex and by age.

Characteristic	All Observations	by Period				by Sex		
	N = 679 ^1^	1–5 Months, N = 355 ^1^	6–9 Months, N = 157 ^1^	≥12 Months, N = 154 ^1^	*p*-Value ^2^	Female, N = 344 ^3^	Male, N = 330 ^3^	*p*-Value ^4^
age	10.0 (6.0, 13.0)/676	9.0 (5.0, 12.0)/353	11.0 (6.0, 13.2)/156	11.0 (6.2, 13.8)/154	0.003	10.0 (6.0, 13.0)/343	10.0 (6.0, 13.0)/328	0.8
Sex					0.7			
female	344 (51%)	181 (51%)	76 (49%)	81 (54%)				
male	330 (49%)	174 (49%)	79 (51%)	70 (46%)				
PICU	4/666 (0.6%)	0/344 (0%)	3/157 (1.9%)	1/152 (0.7%)	0.031	2/338 (0.6%)	2/323 (0.6%)	>0.9
Atopic dermatitis eczema	52/678 (7.7%)	31/355 (8.7%)	8/156 (5.1%)	13/154 (8.4%)	0.4	24/343 (7.0%)	28/330 (8.5%)	0.5
Respiratory diseases	48/678 (7.1%)	29/355 (8.2%)	12/156 (7.7%)	7/154 (4.5%)	0.3	22/343 (6.4%)	26/330 (7.9%)	0.5
Neurological	41/678 (6.0%)	19/355 (5.4%)	10/156 (6.4%)	12/154 (7.8%)	0.6	22/343 (6.4%)	17/330 (5.2%)	0.5
Allergic rhinitis hay fever	25/676 (3.7%)	17/354 (4.8%)	5/155 (3.2%)	1/154 (0.6%)	0.062	8/341 (2.3%)	17/330 (5.2%)	0.055
Anxiety	18/509 (3.5%)	12/257 (4.7%)	3/100 (3.0%)	3/141 (2.1%)	0.4	10/263 (3.8%)	8/243 (3.3%)	0.8
Food allergy	23/678 (3.4%)	13/355 (3.7%)	5/156 (3.2%)	5/154 (3.2%)	>0.9	11/343 (3.2%)	12/330 (3.6%)	0.8
Asthma	21/678 (3.1%)	12/355 (3.4%)	3/156 (1.9%)	6/154 (3.9%)	0.6	8/343 (2.3%)	13/330 (3.9%)	0.2
Gut problems	21/678 (3.1%)	12/355 (3.4%)	5/156 (3.2%)	4/154 (2.6%)	>0.9	11/343 (3.2%)	10/330 (3.0%)	0.9
Excessive weight and obesity	18/678 (2.7%)	11/355 (3.1%)	4/156 (2.6%)	3/154 (1.9%)	0.9	9/343 (2.6%)	9/330 (2.7%)	>0.9
Neurodisability	16/678 (2.4%)	10/355 (2.8%)	1/156 (0.6%)	4/154 (2.6%)	0.3	8/343 (2.3%)	8/330 (2.4%)	>0.9
Skin problems	13/667 (1.9%)	6/351 (1.7%)	3/156 (1.9%)	4/147 (2.7%)	0.7	9/339 (2.7%)	4/323 (1.2%)	0.2
Heart diseases	10/678 (1.5%)	6/355 (1.7%)	2/156 (1.3%)	2/154 (1.3%)	>0.9	5/343 (1.5%)	5/330 (1.5%)	>0.9
Rheumatology	8/677 (1.2%)	3/355 (0.8%)	2/156 (1.3%)	3/154 (1.9%)	0.4	4/342 (1.2%)	4/330 (1.2%)	>0.9
Hematology	8/678 (1.2%)	5/355 (1.4%)	1/156 (0.6%)	2/154 (1.3%)	0.8	7/343 (2.0%)	1/330 (0.3%)	0.069
Immune system diseases	8/678 (1.2%)	5/355 (1.4%)	0/156 (0%)	3/154 (1.9%)	0.3	5/343 (1.5%)	3/330 (0.9%)	0.7
Other	4/656 (0.6%)	3/346 (0.9%)	1/152 (0.7%)	0/146 (0%)	0.8	2/330 (0.6%)	2/321 (0.6%)	>0.9
Genetic conditions	4/678 (0.6%)	2/355 (0.6%)	0/156 (0%)	2/154 (1.3%)	0.4	3/343 (0.9%)	1/330 (0.3%)	0.6
Renal Kidney problems	3/678 (0.4%)	2/355 (0.6%)	0/156 (0%)	1/154 (0.6%)	0.8	0/343 (0%)	3/330 (0.9%)	0.12
Malnutrition	1/675 (0.1%)	1/355 (0.3%)	0/154 (0%)	0/154 (0%)	>0.9	0/341 (0%)	1/329 (0.3%)	0.5
Diabetes	1/677 (0.1%)	0/355 (0%)	1/156 (0.6%)	0/154 (0%)	0.5	0/342 (0%)	1/330 (0.3%)	0.5
Oncology	0/678 (0%)	0/355 (0%)	0/156 (0%)	0/154 (0%)		0/343 (0%)	0/330 (0%)	
Endocrine	0/676 (0%)	0/354 (0%)	0/155 (0%)	0/154 (0%)		0/343 (0%)	0/328 (0%)	
Sickle cell disease	0/287 (0%)	0/160 (0%)	0/24 (0%)	0/92 (0%)		0/142 (0%)	0/145 (0%)	
Depression	0/509 (0%)	0/257 (0%)	0/100 (0%)	0/141 (0%)		0/263 (0%)	0/243 (0%)	
HIV	0/504 (0%)	0/254 (0%)	0/97 (0%)	0/142 (0%)		0/262 (0%)	0/239 (0%)	
TB	0/289 (0%)	0/159 (0%)	0/22 (0%)	0/97 (0%)		0/142 (0%)	0/147 (0%)	

^1^ Median (IQR)/N; n (%); n/N (%). ^2^ Kruskal–Wallis rank sum test; Pearson’s Chi-squared test; Fisher’s exact test. ^3^ Median (IQR)/N; n/N (%). ^4^ Wilcoxon rank sum test; Fisher’s exact test; Pearson’s Chi-squared test. PICU: pediatric intensive care unit; HIV: human immunedeficiency virus; TB: tuberculosis

**Table 2:** Details of persistent symptoms according to the timing of follow-up, sex and age.

Symptom	All Observations	by Period				by Sex			by Age Group		
	N = 679 ^1^	1–5 Months, N = 355 ^1^	6–9 Months, N = 157 ^1^	≥12 Months, N = 154 ^1^	*p*-Value ^2^	Female, N = 344 ^1^	Male, N = 330 ^1^	*p*-Value ^2^	below 10 Years, N = 317 ^1^	≥10 Years, N = 359 ^1^	*p*-Value ^2^
Fatigue	128/679 (19%)	79/355 (22%)	23/157 (15%)	24/154 (16%)	0.062	67/344 (19%)	60/330 (18%)	0.7	37/317 (12%)	89/359 (25%)	<0.001
Headache	82/679 (12%)	49/355 (14%)	14/157 (8.9%)	17/154 (11%)	0.3	53/344 (15%)	29/330 (8.8%)	0.009	17/317 (5.4%)	65/359 (18%)	<0.001
Insomnia	51/679 (7.5%)	33/355 (9.3%)	9/157 (5.7%)	8/154 (5.2%)	0.2	35/344 (10%)	16/330 (4.8%)	0.009	12/317 (3.8%)	39/359 (11%)	<0.001
Persistent muscle pain	47/679 (6.9%)	36/355 (10%)	5/157 (3.2%)	5/154 (3.2%)	0.002	22/344 (6.4%)	25/330 (7.6%)	0.5	14/317 (4.4%)	33/359 (9.2%)	0.015
Confusion lack of concentration	46/678 (6.8%)	30/355 (8.5%)	9/157 (5.7%)	7/153 (4.6%)	0.2	21/343 (6.1%)	25/330 (7.6%)	0.5	14/317 (4.4%)	31/358 (8.7%)	0.027
Stomach abdominal pain	45/678 (6.6%)	29/355 (8.2%)	6/157 (3.8%)	7/154 (4.5%)	0.10	23/344 (6.7%)	22/329 (6.7%)	>0.9	18/317 (5.7%)	27/358 (7.5%)	0.3
Poor appetite	43/679 (6.3%)	22/355 (6.2%)	14/157 (8.9%)	7/154 (4.5%)	0.3	19/344 (5.5%)	24/330 (7.3%)	0.4	19/317 (6.0%)	24/359 (6.7%)	0.7
Skin rash	33/678 (4.9%)	16/355 (4.5%)	12/156 (7.7%)	4/154 (2.6%)	0.10	17/344 (4.9%)	16/329 (4.9%)	>0.9	13/316 (4.1%)	20/359 (5.6%)	0.4
Persistent cough	31/679 (4.6%)	20/355 (5.6%)	5/157 (3.2%)	5/154 (3.2%)	0.3	8/344 (2.3%)	23/330 (7.0%)	0.004	21/317 (6.6%)	10/359 (2.8%)	0.017
Joint pain or swelling	30/679 (4.4%)	20/355 (5.6%)	4/157 (2.5%)	4/154 (2.6%)	0.15	16/344 (4.7%)	14/330 (4.2%)	0.8	7/317 (2.2%)	23/359 (6.4%)	0.008
Diarrhea	30/679 (4.4%)	16/355 (4.5%)	4/157 (2.5%)	8/154 (5.2%)	0.5	9/344 (2.6%)	21/330 (6.4%)	0.018	10/317 (3.2%)	20/359 (5.6%)	0.13
Constipation	30/679 (4.4%)	17/355 (4.8%)	8/157 (5.1%)	5/154 (3.2%)	0.7	14/344 (4.1%)	14/330 (4.2%)	>0.9	22/317 (6.9%)	8/359 (2.2%)	0.003
Chest pain	26/679 (3.8%)	12/355 (3.4%)	10/157 (6.4%)	0/154 (0%)	0.007	14/344 (4.1%)	12/330 (3.6%)	0.8	5/317 (1.6%)	21/359 (5.8%)	0.004
Weight loss	16/436 (3.7%)	9/213 (4.2%)	5/141 (3.5%)	2/77 (2.6%)	>0.9	8/223 (3.6%)	8/208 (3.8%)	0.9	3/175 (1.7%)	13/261 (5.0%)	0.075
Difficulty breathing chest tightness	20/679 (2.9%)	14/355 (3.9%)	3/157 (1.9%)	0/154 (0%)	0.017	8/344 (2.3%)	12/330 (3.6%)	0.3	7/317 (2.2%)	13/359 (3.6%)	0.3
Feeling nauseous	19/679 (2.8%)	14/355 (3.9%)	3/157 (1.9%)	1/154 (0.6%)	0.086	10/344 (2.9%)	9/330 (2.7%)	0.9	3/317 (0.9%)	16/359 (4.5%)	0.006
Alternated sense of smell	17/679 (2.5%)	8/355 (2.3%)	5/157 (3.2%)	3/154 (1.9%)	0.8	9/344 (2.6%)	8/330 (2.4%)	0.9	1/317 (0.3%)	16/359 (4.5%)	<0.001
Alternated taste	17/679 (2.5%)	11/355 (3.1%)	4/157 (2.5%)	2/154 (1.3%)	0.6	11/344 (3.2%)	6/330 (1.8%)	0.3	1/317 (0.3%)	16/359 (4.5%)	<0.001
Pain on breathing	15/604 (2.5%)	8/326 (2.5%)	4/151 (2.6%)	0/116 (0%)	0.2	8/302 (2.6%)	7/297 (2.4%)	0.8	3/281 (1.1%)	12/320 (3.8%)	0.035
Hypersomnia	15/679 (2.2%)	11/355 (3.1%)	1/157 (0.6%)	3/154 (1.9%)	0.2	8/344 (2.3%)	7/330 (2.1%)	0.9	4/317 (1.3%)	11/359 (3.1%)	0.11
Loss of smell	11/679 (1.6%)	5/355 (1.4%)	2/157 (1.3%)	3/154 (1.9%)	0.8	6/344 (1.7%)	5/330 (1.5%)	0.8	1/317 (0.3%)	10/359 (2.8%)	0.011
Loss of taste	11/679 (1.6%)	7/355 (2.0%)	1/157 (0.6%)	3/154 (1.9%)	0.6	5/344 (1.5%)	6/330 (1.8%)	0.7	3/317 (0.9%)	8/359 (2.2%)	0.2
Feeling sick vomiting	8/679 (1.2%)	5/355 (1.4%)	1/157 (0.6%)	1/154 (0.6%)	0.8	3/344 (0.9%)	5/330 (1.5%)	0.5	4/317 (1.3%)	4/359 (1.1%)	>0.9
Problems speaking or communicating	3/443 (0.7%)	2/211 (0.9%)	0/142 (0%)	1/87 (1.1%)	0.4	1/230 (0.4%)	2/208 (1.0%)	0.6	2/174 (1.1%)	1/268 (0.4%)	0.6
Fainting blackouts	4/679 (0.6%)	1/355 (0.3%)	1/157 (0.6%)	1/154 (0.6%)	0.6	4/344 (1.2%)	0/330 (0%)	0.12	1/317 (0.3%)	3/359 (0.8%)	0.6
Tremor shakiness	2/490 (0.4%)	2/257 (0.8%)	0/139 (0%)	0/86 (0%)	0.7	1/253 (0.4%)	1/232 (0.4%)	>0.9	1/204 (0.5%)	1/286 (0.3%)	>0.9
Tingling feeling pins and needles	2/679 (0.3%)	1/355 (0.3%)	1/157 (0.6%)	0/154 (0%)	0.7	2/344 (0.6%)	0/330 (0%)	0.5	1/317 (0.3%)	1/359 (0.3%)	>0.9
Cannot fully move or control movements	1/412 (0.2%)	1/201 (0.5%)	0/137 (0%)	0/71 (0%)	>0.9	0/217 (0%)	1/190 (0.5%)	0.5	0/161 (0%)	1/251 (0.4%)	>0.9
Bilateral conjunctivitis	1/415 (0.2%)	0/207 (0%)	1/136 (0.7%)	0/71 (0%)	0.5	1/217 (0.5%)	0/193 (0%)	>0.9	1/163 (0.6%)	0/252 (0%)	0.4
Seizures fits	1/432 (0.2%)	1/212 (0.5%)	0/137 (0%)	0/81 (0%)	>0.9	1/225 (0.4%)	0/202 (0%)	>0.9	1/173 (0.6%)	0/259 (0%)	0.4
Problems with balance	1/679 (0.1%)	0/355 (0%)	0/157 (0%)	0/154 (0%)		1/344 (0.3%)	0/330 (0%)	>0.9	0/317 (0%)	1/359 (0.3%)	>0.9
Dizziness light headedness	1/679 (0.1%)	1/355 (0.3%)	0/157 (0%)	0/154 (0%)	>0.9	0/344 (0%)	1/330 (0.3%)	0.5	0/317 (0%)	1/359 (0.3%)	>0.9
Double vision blurred vision	0/679 (0%)	0/355 (0%)	0/157 (0%)	0/154 (0%)		0/344 (0%)	0/330 (0%)		0/317 (0%)	0/359 (0%)	
Problems swallowing or chewing	0/411 (0%)	0/196 (0%)	0/137 (0%)	0/76 (0%)		0/212 (0%)	0/194 (0%)		0/159 (0%)	0/252 (0%)	
purulent non purulent	0/148 (0%)	0/69 (0%)	0/61 (0%)	0/17 (0%)		0/75 (0%)	0/72 (0%)		0/61 (0%)	0/87 (0%)	
Lumps or rashes on toes	0/394 (0%)	0/196 (0%)	0/135 (0%)	0/62 (0%)		0/206 (0%)	0/183 (0%)		0/156 (0%)	0/238 (0%)	

^1^ evaluated at that follow-up time; ^2^ Pearson’s Chi-squared test; Fisher’s exact test.

**Table 3:** Persistent symptoms comparing Omicron and original variants at 1-5 months’ follow-up. Only the children followed up within the same time period were included.

Characteristic	Omicron, N = 120 ^1^	Wild Type, N = 201 ^1^	*p*-Value ^2^
Fatigue	16/120 (13%)	50/201 (25%)	0.013
Headache	11/120 (9.2%)	31/201 (15%)	0.11
Insomnia	6/120 (5.0%)	27/201 (13%)	0.016
Persistent muscle pain	4/120 (3.3%)	27/201 (13%)	0.003
Confusion lack of concentration	10/120 (8.3%)	18/201 (9.0%)	0.8
Stomach abdominal pain	6/120 (5.0%)	17/201 (8.5%)	0.2
Poor appetite	4/120 (3.3%)	17/201 (8.5%)	0.072
Skin rash	3/120 (2.5%)	11/201 (5.5%)	0.2
Persistent cough	11/120 (9.2%)	6/201 (3.0%)	0.017
Joint pain or swelling	0/120 (0%)	17/201 (8.5%)	0.001
Diarrhea	4/120 (3.3%)	10/201 (5.0%)	0.5
Constipation	3/120 (2.5%)	14/201 (7.0%)	0.084
Chest pain	2/120 (1.7%)	9/201 (4.5%)	0.2
Weight loss	0/10 (0%)	9/196 (4.6%)	>0.9
Difficulty breathing chest tightness	2/120 (1.7%)	9/201 (4.5%)	0.2
Feeling nauseous	3/120 (2.5%)	10/201 (5.0%)	0.4
Alternated sense of smell	0/120 (0%)	6/201 (3.0%)	0.088
Alternated taste	0/120 (0%)	8/201 (4.0%)	0.027
Pain on breathing	0/99 (0%)	7/200 (3.5%)	0.10

^1^ n/N (%). ^2^ Pearson’s Chi-squared test; Fisher’s exact test.

**Table 4:** Self-reported recovery rates according to time of follow-up, sex and age.

	All Observations	by Period				by Sex			by Age Group		
Characteristic	N = 679 ^1^	1–5 Months, N = 355 ^1^	6–9 Months, N = 157 ^1^	12 Months and More, N = 154 ^1^	*p*-Value ^2^	Female, N = 344 ^1^	Male, N = 330 ^1^	*p*-Value ^3^	below 10 Years, N = 317 ^1^	10 Years and above, N = 359 ^1^	*p*-Value ^3^
Fully recovered					0.003			0.014			0.047
1–4	17 (2.6%)	14 (4.0%)	2 (1.3%)	1 (0.7%)		3 (0.9%)	14 (4.4%)		3 (1.0%)	13 (3.7%)	
5–7	73 (11%)	46 (13%)	7 (4.6%)	15 (9.9%)		35 (10%)	38 (12%)		31 (9.9%)	41 (12%)	
8–10	576 (86%)	288 (83%)	144 (94%)	136 (89%)		302 (89%)	269 (84%)		279 (89%)	296 (85%)	

^1^ n (%). ^2^ Fisher’s exact test. ^3^ Pearson’s Chi-squared test.

**Table 5:** Recovery dynamics for patients with repeated follow-ups.

fup_Period_1	fup_Period_2	Values_1	Values_2	*p*-Value
1–5	6–9	14/143	0/143	0.000
1–5	≥12 m	14/142	7/142	0.174
6–9	≥12 m	2/121	3/121	1.000

## Data Availability

The dataset is available upon request from the corresponding author.

## Supplementary Materials

The following supporting information can be downloaded at: https://www.mdpi.com/article/10.3390/jcm11226772/s1, Table S1: Number of persistent symptoms according to time of follow-up, gender and age. Table S2: Details of recovery rates according to age and sex and follow-up. Table S3: Recovery rates comparing Omicron and the wild virus. Table S4: Neurocognitive issues reported. Table S5: Wellbeing reported. Figure S1: The pattern of persistent symptoms is more common in girls than boys. Figure S2: The pattern of persistent symptoms is more common in children younger or older than 10 years of age. Supplementary Methods: the CRF.
