# Supplementary material for: The Prevalence, Characteristics and Risk Factors of Persistent Symptoms in Non-Hospitalized and Hospitalized Children with SARS-CoV-2 Infection Followed-Up for up to 12 Months: A Prospective, Cohort Study in Rome, Italy
Source: J Clin Med. 2022 Nov 16;11(22):6772. doi: 10.3390/jcm11226772 (PMC9692851; doi:10.3390/jcm11226772)
Supplement: Supplementary file 1 [file jcm-11-06772-s001.zip › jcm-2006365-supplementary.pdf]

Supplementary material

Symptom	All observations	By period				By sex			By age group		
N = 679[1]		1 - 5 months, N = 355[1]	6 - 9 months, N = 157[1]	12 months and more, N = 154[1]	p-value[2]	female, N = 344[1]	male, N = 330[1]	p-value[2]	below 10 years, N = 317[1]	10 years and above, N = 359[1]	p-value[2]
Number of symptoms reported					0.024			0.7			<0.001
No persisting symptoms reported	352 (52%)	164 (46%)	88 (56%)	97 (63%)		172 (50%)	178 (54%)		194 (61%)	157 (44%)	
Only one persisting symptom reported	160 (24%)	95 (27%)	32 (20%)	30 (19%)		83 (24%)	74 (22%)		78 (25%)	81 (23%)	
Two persisting symptoms reported	60 (8.8%)	32 (9.0%)	15 (9.6%)	11 (7.1%)		30 (8.7%)	30 (9.1%)		20 (6.3%)	39 (11%)	
Three or more persisting symptoms reported	107 (16%)	64 (18%)	22 (14%)	16 (10%)		59 (17%)	48 (15%)		25 (7.9%)	82 (23%)	
[1] n (%)											
[2] Pearson's Chi-squared test											

Supplementary table S1. Number of persisting symptoms according to time of follow-up, gender and age.

Population_category	1-4	5-7	8-10
1 - 5 months / female / 10 years and above	3 (3.5%)	12 (14.0%)	71 (82.6%)
1 - 5 months / female / below 10 years		7 (7.5%)	86 (92.5%)
1 - 5 months / male / 10 years and above	7 (9.5%)	9 (12.2%)	58 (78.4%)
1 - 5 months / male / below 10 years	3 (3.2%)	17 (18.3%)	73 (78.5%)
6 - 9 months / female / 10 years and above		4 (9.1%)	40 (90.9%)
6 - 9 months / female / below 10 years			30 (100.0%)
6 - 9 months / male / 10 years and above	2 (4.5%)	1 (2.3%)	41 (93.2%)
6 - 9 months / male / below 10 years		2 (6.2%)	30 (93.8%)
12 months and more / female / 10 years and above		8 (16.0%)	42 (84.0%)
12 months and more / female / below 10 years		2 (6.7%)	28 (93.3%)
12 months and more / male / 10 years and above	1 (2.4%)	4 (9.8%)	36 (87.8%)
12 months and more / male / below 10 years		1 (3.6%)	27 (96.4%)

Table S2. Details of recovery rates according to age and sex and follow-up.

Characteristic	Omicron, N = 120[1]	unspecified, N = 201[1]	p-value[2]
Fully recovered			0.12
1-4	3 (2.5%)	9 (4.6%)	
5-7	19 (16%)	17 (8.7%)	
8-10	97 (82%)	169 (87%)	

[1]n (%)

[2]Fisher's exact test

Table S3. Recovery rate comparing Omicron and wild virus

	All observations	By period				By sex			By age group		
Characteristic	N = 658[1]	1 - 5 months, N = 353[1]	6 - 9 months, N = 154[1]	12 months and more, N = 151[1]	p-value[2]	female, N = 337[1]	male, N = 321[1]	p-value[2]	below 10 years, N = 310[1]	10 years and above, N = 348[1]	p-value[2]
Tiredness	64 / 304 (21%)	41 / 156 (26%)	6 / 22 (27%)	17 / 126 (13%)	0.025	25 / 153 (16%)	39 / 151 (26%)	0.042	29 / 172 (17%)	35 / 132 (27%)	0.041
Rest more	67 / 304 (22%)	45 / 156 (29%)	7 / 22 (32%)	15 / 126 (12%)	0.002	25 / 153 (16%)	42 / 151 (28%)	0.016	33 / 172 (19%)	34 / 132 (26%)	0.2
Sleepy or drowsy	33 / 304 (11%)	18 / 156 (12%)	3 / 22 (14%)	12 / 126 (9.5%)	0.8	15 / 153 (9.8%)	18 / 151 (12%)	0.6	10 / 172 (5.8%)	23 / 132 (17%)	0.001
Problems starting things	18 / 305 (5.9%)	9 / 157 (5.7%)	1 / 22 (4.5%)	8 / 126 (6.3%)	>0.9	8 / 154 (5.2%)	10 / 151 (6.6%)	0.6	7 / 173 (4.0%)	11 / 132 (8.3%)	0.12
Lack energy	68 / 306 (22%)	43 / 157 (27%)	7 / 22 (32%)	18 / 127 (14%)	0.015	28 / 155 (18%)	40 / 151 (26%)	0.076	32 / 174 (18%)	36 / 132 (27%)	0.064
Less strength in your muscles	63 / 306 (21%)	37 / 157 (24%)	8 / 22 (36%)	18 / 127 (14%)	0.025	27 / 155 (17%)	36 / 151 (24%)	0.2	28 / 174 (16%)	35 / 132 (27%)	0.026
Feel weak	76 / 306 (25%)	47 / 157 (30%)	8 / 22 (36%)	21 / 127 (17%)	0.015	32 / 155 (21%)	44 / 151 (29%)	0.086	37 / 174 (21%)	39 / 132 (30%)	0.10
Difficulties concentrating	42 / 304 (14%)	25 / 156 (16%)	3 / 22 (14%)	14 / 126 (11%)	0.5	20 / 153 (13%)	22 / 151 (15%)	0.7	17 / 172 (9.9%)	25 / 132 (19%)	0.023
Slips of the tongue when speaking	12 / 304 (3.9%)	5 / 156 (3.2%)	2 / 22 (9.1%)	5 / 126 (4.0%)	0.3	4 / 153 (2.6%)	8 / 151 (5.3%)	0.2	5 / 172 (2.9%)	7 / 132 (5.3%)	0.3
Difficulty finding the right word	16 / 304 (5.3%)	8 / 156 (5.1%)	3 / 22 (14%)	5 / 126 (4.0%)	0.15	8 / 153 (5.2%)	8 / 151 (5.3%)	>0.9	7 / 172 (4.1%)	9 / 132 (6.8%)	0.3
Memory	10 / 303 (3.3%)	5 / 156 (3.2%)	2 / 22 (9.1%)	3 / 125 (2.4%)	0.2	5 / 153 (3.3%)	5 / 150 (3.3%)	>0.9	5 / 172 (2.9%)	5 / 131 (3.8%)	0.8

[1]n / N (%)

[2]Pearson's Chi-squared test; Fisher's exact test

Table S4. Neurocognitive issues reported

Characteristic	All observations	By period				By sex			By age group		
	N = 658[1]	1 - 5 months, N = 353[1]	6 - 9 months, N = 154[1]	12 months and more, N = 151[1]	p-value[2]	female, N = 337[1]	male, N = 321[1]	p-value[3]	below 10 years, N = 310[1]	10 years and above, N = 348[1]	p-value[3]
Eating	70 / 656 (11%)	44 / 353 (12%)	17 / 154 (11%)	9 / 149 (6.0%)	0.10	34 / 336 (10%)	36 / 320 (11%)	0.6	33 / 308 (11%)	37 / 348 (11%)	>0.9
Sleeping	92 / 656 (14%)	56 / 353 (16%)	25 / 154 (16%)	11 / 149 (7.4%)	0.029	52 / 336 (15%)	40 / 320 (12%)	0.3	33 / 308 (11%)	59 / 348 (17%)	0.022
Physical activity	238 / 656 (36%)	149 / 353 (42%)	58 / 154 (38%)	31 / 149 (21%)	<0.001	123 / 336 (37%)	115 / 320 (36%)	0.9	86 / 308 (28%)	152 / 348 (44%)	<0.001
More fatigue	147 / 656 (22%)	97 / 353 (27%)	25 / 154 (16%)	25 / 149 (17%)	0.003	76 / 336 (23%)	71 / 320 (22%)	0.9	54 / 308 (18%)	93 / 348 (27%)	0.005
Time spent with friends in person	180 / 615 (29%)	97 / 329 (29%)	53 / 148 (36%)	30 / 138 (22%)	0.033	90 / 315 (29%)	90 / 300 (30%)	0.7	60 / 284 (21%)	120 / 331 (36%)	<0.001
Time spent with friends remotely	72 / 617 (12%)	41 / 332 (12%)	24 / 147 (16%)	7 / 138 (5.1%)	0.011	32 / 318 (10%)	40 / 299 (13%)	0.2	16 / 284 (5.6%)	56 / 333 (17%)	<0.001
Spending time outside	193 / 618 (31%)	106 / 331 (32%)	62 / 148 (42%)	25 / 139 (18%)	<0.001	96 / 318 (30%)	97 / 300 (32%)	0.6	76 / 287 (26%)	117 / 331 (35%)	0.018
more screen time for non educational purposes	127 / 613 (21%)	78 / 329 (24%)	36 / 146 (25%)	13 / 138 (9.4%)	<0.001	56 / 315 (18%)	71 / 298 (24%)	0.065	40 / 282 (14%)	87 / 331 (26%)	<0.001
Attending school	99 / 607 (16%)	59 / 327 (18%)	20 / 145 (14%)	20 / 135 (15%)	0.4	39 / 313 (12%)	60 / 294 (20%)	0.008	35 / 277 (13%)	64 / 330 (19%)	0.025

Connectedness	12 / 611 (2.0%)	5 / 328 (1.5%)	7 / 146 (4.8%)	0 / 137 (0%)	0.009	6 / 316 (1.9%)	6 / 295 (2.0%)	>0.9	5 / 281 (1.8%)	7 / 330 (2.1%)	0.8
Emotions	161 / 656 (25%)	86 / 353 (24%)	51 / 154 (33%)	24 / 149 (16%)	0.003	83 / 336 (25%)	78 / 320 (24%)	>0.9	53 / 308 (17%)	108 / 348 (31%)	<0.001
Behaviour	116 / 654 (18%)	59 / 353 (17%)	33 / 153 (22%)	24 / 148 (16%)	0.4	57 / 336 (17%)	59 / 318 (19%)	0.6	60 / 308 (19%)	56 / 346 (16%)	0.3
Relationships	56 / 611 (9.2%)	21 / 327 (6.4%)	26 / 146 (18%)	9 / 138 (6.5%)	<0.001	25 / 319 (7.8%)	31 / 292 (11%)	0.2	17 / 281 (6.0%)	39 / 330 (12%)	0.014

[1] n / N (%)

[2] Pearson's Chi-squared test; Fisher's exact test

[3] Pearson's Chi-squared test

Table S5. Wellbeing reported reported

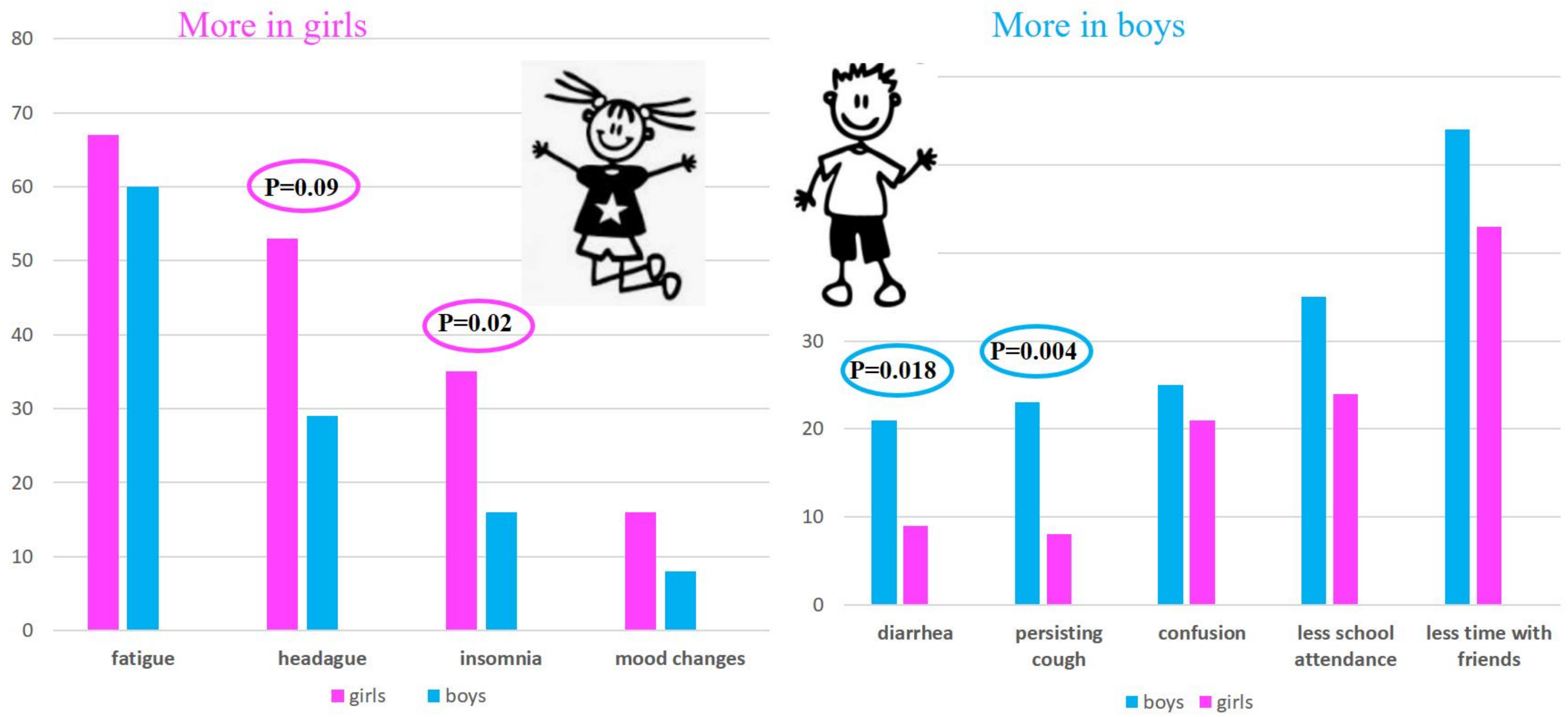

Supplementary figure S1: pattern of persisting symptoms more common in girls or boys

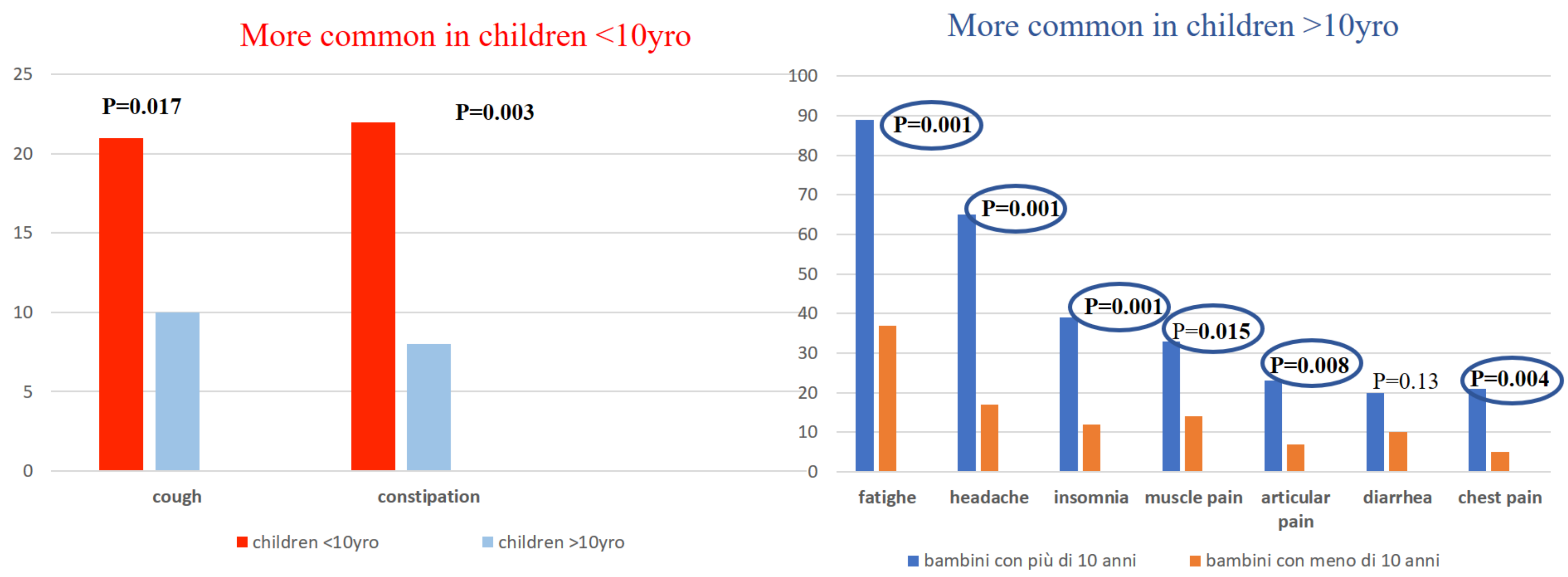

Supplementary figure S2: pattern of persisting symptoms more common in children younger or older than 10 years of age

Supplementary methods

Annexes

COVID-19 Health and Wellbeing **Initial** Follow Up Survey for Children, according to the ISARIC proposal

PARENT REPORT FOR CHILDREN AND YOUNG PEOPLE

(between 0 and 18 years of age)

SURVEY TIMEPOINT (to be completed by the team before sending or administering the survey): 3m [_____] 6m [_] 12m [______] 24m [__] 36m [__]

Survey completed: □ Online □Telephone □Clinic led assessment

□ Mother/female caregiver □ Father/male caregiver □ Other If *other*, please specify _________________

Your permission to proceed

Thank you for coming this far. Now to take part, please read the statements below, and initial the boxes if you are happy to go ahead.

PLEASE MARK YOUR INITIALS AGAINST EACH STATEMENT WITH WHICH YOU AGREE:	*Initials:*	
I give my consent for the information I provide in this study to be used as advised.		

I would like to continue to be sent this survey via email, post or to be contacted via telephone follow up every 3 to 6 months for a maximum of 3 years after my child's Covid-19 illness.<br><br>If yes, please enter your contact details here:<br>E-mail:____<br>_____________________________________<br>Mobile phone number: ____<br>Home telephone number: ____	YE<br>S	NO
I would like the possibility to be contacted by a nurse, doctor or researcher to discuss my child's Covid-19 illness further.<br><br>If yes, please enter your contact details here:<br>Mobile phone number: _______________________<br>Home telephone number: ____	YE<br>S	NO

You are completing this survey on behalf of your child, please enter your and their details

Your child's first name:________________Surname:

Your first name: ______________________Surname:____________________

Town/City of residence:_________________Postcode:

Your signature:____

Local hospital ID:____

PARTICIPANT IDENTIFICATION# : [__][_____][_____][_____][_____

1a. About you child

Sex assigned at Birth: □ Male □ Female □ Prefer not to say

Ethnicity (tick all that apply): □ White □ Arab □ Black □ East Asian □ South Asian

□ West Asian □ Latin American □Other (specify):____________□ Prefer not to say

What is your child's estimated height: _______(□ metres □ feet/inches) □ Not sure

What is your child's current estimated weight: __________(□ kg □ lbs □ stone) □ Not sure

What was your child's estimated weight before Covid19 illness? ___________ (□ kg □ lbs□ stone) □ Not sure

How many other members regularly live in your household, including yourself: [_Number_]

1b. About <u>your child's</u> Covid-19 illness - all the questions relate to his/her health and wellbeing)

Date you completed the survey (DD/MM/YYYY):[_D_][_D_]/[_M_][_M_]/[_2_][_0_][_Y_][_Y_]

What is your child date of birth (DD/MM/YYYY):[_D_][_D_]/[_M_][_M_]/[_Y_][_Y_][_Y_][_Y_]

Approximately, what day did you first notice your child was experiencing symptoms of Covid-19? [_D_][_D_]/[_M_][_M_]/[_2_][_0_][_Y_][_Y_]

How was your child diagnosed with Covid-19?

□ Laboratory confirmed (PCR or/and Antibody test)

□ Physician confirmed (no laboratory testing was performed) □ Not sure

Has your child been admitted to hospital due to Covid-19? □ Yes □ No

(If the answer is "no", please, move on to the section "2"; if the answer is "yes", please, proceed with the following questions)

- **Roughly at what date was your child first admitted to hospital?** [_D_][_D_]/[_M_][_M_]/[_2_][_0_][_Y_][_Y_]
- **Roughly at what date was your child first discharged from hospital?** [_D_][_D_]/[_M_][_M_]/[_2_][_0_][_Y_][_Y_]
- **Spent any time in the Paediatric Intensive Care Unit (PICU)?** □ Yes □ No □ Not sure
- **Hospital admission or health facility visit after Covid-19 illness?** □ Yes □ No

If yes, how many times: [_Number_]

If yes, specify reason/reasons: ____

Name of hospital/hospitals: ____

2a. About your child's emotional wellbeing, social relationships and activities'

To answer the following questions, please **mark an X** on the lines below that shows your opinion on the question. In each scale, **1 is the least favorable value, 5 is the most favorable value.**

1. **Compared to before the Covid-19 infection, how much is the child now doing the following**
If there are changes, please indicate whether you think these are due to the illness itself or to the Covid-19 pandemic (e.g. changes in social activities)

Eating

1 2 3 4 5
Less The same More

If there are changes, *please indicate whether you think these are due to*
□ *Illness itself* □ *Covid-19 pandemic* □ *Both* □ *Unsure*
□ *Unsure*

Sleeping

1 2 3 4 5
Less The same More

If there are changes, *please indicate whether you think these are due to*
□ *Illness itself* □ *Covid-19 pandemic* □ *Both*

Physical activity

1 2 3 4 5
Less The same More

If there are changes, *please indicate whether you think these are due to*
□ *Illness itself* □ *Covid-19 pandemic* □ *Both* □ *Unsure*
□ *Unsure*

Fatigue

1 2 3 4 5
Less The same More

If there are changes, *please indicate whether you think these are due to*
□ *Illness itself* □ *Covid-19 pandemic* □ *Both*

Spending time with friends in-person

Spending time with friends remotely (e.g., online, social media, texting)

1 2 3 4 5
Less The same More

If there are changes, *please indicate whether you think these are due to*
□ Illness itself □ Covid-19 pandemic □ Both □ Unsure

1 2 3 4 5
Less The same More

If there are changes, *please indicate whether you think these are due to*
□ Illness itself □ Covid-19 pandemic □ Both □ Unsure

Spending time outside

1 2 3 4 5
Less The same More

If there are changes, please indicate whether you think these are due to
□ Illness itself □ Covid-19 pandemic □ Both □ Unsure

Spending time watching TV, playing video/computer games, or using social media for educational purposes, including school work

1 2 3 4 5
Less The same More

If there are changes, *please indicate whether you think these are due to*
□ Illness itself □ Covid-19 pandemic □ Both □ Unsure

Spending time watching TV, playing video/computer games, or using social media for non-educational purposes

1 2 3 4 5
Less The same More

If there are changes, *please indicate whether you think these are due to*
□ Illness itself □ Covid-19 pandemic □ Both □ Unsure

Attending school

1 2 3 4 5
Less The same More

If there are changes, please indicate whether you think these are due to
□ Illness itself □ Covid-19 pandemic □ Both □ Unsure

2. **Compared to before the Covid-19 infection: Have there been changes in your child's CONNECTEDNESS with others since…**

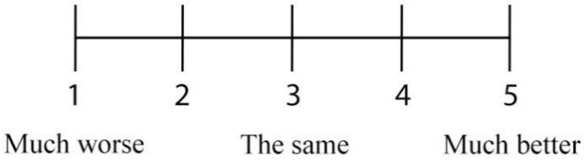

If there are changes, please indicate whether you think these are due to
□ Illness itself □ Covid-19 pandemic □ Both □ Unsure

3. **Compared to before the Covid-19 infection have there been any changes in your child's EMOTIONS?**

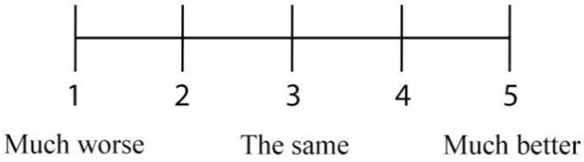

If there are changes, please indicate whether you think these are due to
□ Illness itself □ Covid-19 pandemic □ Both □ Unsure

4. **Compared to before the Covid-19 infection have there been any changes in your child's BEHAVIOUR?**

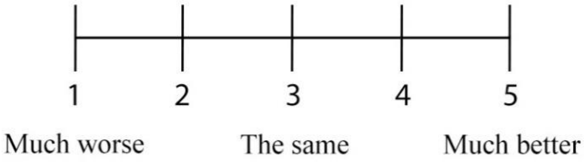

If there are changes, please indicate whether you think these are due to
□ Illness itself □ Covid-19 pandemic □ Both □ Unsure

5. **Compared to before the Covid-19 infection have there been any changes in your child's RELATIONSHIPS, in how they get on with others?**

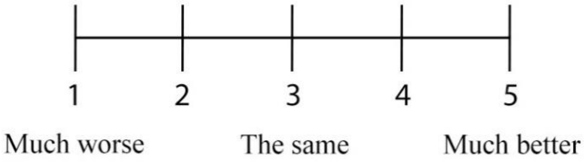

If there are changes, please indicate whether you think these are due to
□ Illness itself □ Covid-19 pandemic □ Both □ Unsure

6. **Have you asked for help from a health professional because of Covid-19 consequences to your child's EMOTIONS, BEHAVIOUR OR RELATIONSHIPS?** □ Yes □ No

If so, who did you ask help from________________________________

If you have replied MUCH WORSE to any of the above OR YOU HAVE ASKED FOR HELP for these problems:

• Do the difficulties upset or distress your child?
□ Not at all □ Only a little □ Quite a lot □ A great deal

• Do the difficulties interfere with your child's everyday life in the following areas?

Home Life	□ Not at all □ Only a little □ Quite a lot □ A great deal
Friendships	□ Not at all □ Only a little □ Quite a lot □ A great deal
Classroom Learning	□ Not at all □ Only a little □ Quite a lot □ A great deal
Leisure Activities	□ Not at all □ Only a little □ Quite a lot □ A great deal

• Do the difficulties put a burden on you or the family as a whole?
□ Not at all □ Only a little □ Quite a lot □ A great deal

7. What is the current situation in your town/city/region on lockdown measures…?

□ Closing of schools and kindergartens

□ Closing of non-essential shops (shops and stores apart from food, doctors and drug stores)

□ Closing of non-essential production

□ Cancellation of recreational venues and closing of public places

□ Constraining meeting friends

□ Curfews

□ Stay-at-home orders and/or total movement control

2b. About your child's current state of health

Has your child RECEIVED TREATMENT for any of the following chronic medical conditions prior to the Covid-19 infection?

	Yes	No	Unknown
Prematurity *(baby born <37 weeks)*	□	□	□
Neurological *(pertaining to the nervous system)*	□	□	□
Neurodisability	□	□	□
Heart diseases	□	□	□
Respiratory diseases (not including asthma)	□	□	□
Asthma (doctor's diagnosed)	□	□	□
Allergic rhinitis/hay fever	□	□	□
Food allergy	□	□	□
Atopic dermatitis/Eczema	□	□	□
Skin problems (not including eczema)	□	□	□
Gut problems	□	□	□
Haematology *(blood diseases)*	□	□	□
Oncology *(cancer or other progressively enlarging and spreading tumor)*	□	□	□
Immune system diseases	□	□	□
Genetic conditions	□	□	□
Diabetes	□	□	□
Endocrine (not diabetes)	□	□	□
Renal/Kidney problems	□	□	□
Excessive weight and obesity	□	□	□
Malnutrition *(deficiencies, excesses, or imbalances in a person's intake of energy and/or nutrients)*	□	□	□

Rheumatology *(e.g. arthritis, or inflammation of the joints)*	☐	☐	☐
Other (please indicate)			

Prior to COVID-19 infection, how was your child's health in general?

☐ Very bad ☐ Bad ☐ Fair ☐ Good ☐ Very good

If you ticked bad or very bad, please explain:

Prior to COVID-19 infection, how would you describe your child's mental health in general

☐ Very bad ☐ Bad ☐ Fair ☐ Good ☐ Very good

If you ticked bad or very bad, please explain:

Has your child ever been under Child and Adolescent Mental Health services before Covid-19 pandemic? ☐ Yes ☐ No ☐ Not sure

Have you requested help because of Covid-19 consequences to your child's physical health? ☐ ☐ Yes ☐ No ☐ Not sure

Has your child felt feverish recently? ☐ Yes ☐ No ☐ Not sure

(If the answer is "no", please, move on to the next question)

☐ Within the last 7 days ☐ 1-2 weeks ☐ >2-4 weeks ☐ >1-2 months ☐ >2-3 months ☐ >3-6 months

☐ Since discharge

If yes, what was the cause of your child's most recent feverish illness?

☐ Covid-19 ☐ Other respiratory infection (cough/cold/sore throat) ☐ TB

☐ Stomach infection (diarrhea/vomiting) ☐ Urinary infection

☐ Other: specify: ___________________________________

☐ Unknown ☐ Prefer not to say

Has your child been reinfected with Covid-19? ☐ Yes ☐ No

If yes, what were the recent laboratory results for SARS-CoV-2? ______________________

Date of last positive SARS-CoV-2 test [_D_][_D_]/[_M_][_M_]/[_2_][_0_][_Y_][_Y_]

How much do you agree with the following statement?

"My child has fully recovered from Covid-19"

Please **mark an X** on the line below that shows your opinion on the question as of **TODAY**

0 1 2 3 4 5 6 7 8 9 10

Strongly disagree Neutral Strongly agree

3. Since having Covid-19, has your child been diagnosed with any of the following?

Multisystem inflammatory syndrome	□ Yes	□ No	Shock / Toxic shock syndrome	□ Yes	□ No
Pulmonary embolism<br>*(PE, "Clot in lung")*	□ Yes	□ No	Coagulopathy<br>*(excessive bleeding or clotting)*	□ Yes	□ No
Kawasaki disease	□ Yes	□ No	Kidney problems	□ Yes	□ No
Respiratory failure	□ Yes	□ No	Type 1 Diabetes	□ Yes	□ No
Myocarditis<br>*(inflammation of the heart muscle)*	□ Yes	□ No	Type 2 Diabetes	□ Yes	□ No
Asthma	□ Yes	□ No	Intussusception	□ Yes	□ No

Other condition (please specify)?

____

4. Within the <u>last seven days</u>, have you had any of these symptoms, which were NOT present prior to Covid-19? If yes, please indicate duration of the symptom(-s).

**Respiratory problems**	**Yes/No**	**<u>If yes</u>**, what is the duration of symptoms
Nasal congestion / rhinorrhea	□ Yes □ No	□ less than 1 month □ 1-3 months □ over 3 to 6 months □ more than 6 months
Difficulty breathing /chest tightness	□ Yes □ No	□ less than 1 month □ 1-3 months □ over 3 to 6 months □ more than 6 months
Pain on breathing	□ Yes □ No	□ less than 1 month □ 1-3 months □ over 3 to 6 months □ more than 6 months
Chest pain	□ Yes □ No	□ less than 1 month □ 1-3 months □ over 3 to 6 months □ more than 6 months
Persistent cough	□ Yes □ No	□ less than 1 month □ 1-3 months □ over 3 to 6 months □ more than 6 months
If yes, □ *dry cough* □ *with phlegm*		

**Musculoskeletal problems**	**Yes/No**	**<u>If yes</u>**, what is the duration of symptoms
Cannot fully move or control movement	□ Yes □ No	□ less than 1 month □ 1-3 months □ over 3 to 6 months □ more than 6 months
Problems with balance	□ Yes □ No	□ less than 1 month □ 1-3 months □ over 3 to 6 months □ more than 6 months
Persistent muscle pain	□ Yes □ No	□ less than 1 month □ 1-3 months □ over 3 to 6 months □ more than 6 months
Joint pain or swelling	□ Yes □ No	□ less than 1 month □ 1-3 months □ over 3 to 6 months □ more than 6 months

**Neurological problems**	**Yes/No**	**<u>If yes</u>**, what is the duration of symptoms
Headache	□ Yes □ No	□ less than 1 month □ 1-3 months □ over 3 to 6 months □ more than 6 months
Dizziness/ light headedness	□ Yes □ No	□ less than 1 month □ 1-3 months □ over 3 to 6 months □ more than 6 months
Fainting/ blackouts	□ Yes □ No	□ less than 1 month □ 1-3 months □ over 3 to 6 months □ more than 6 months
Double vision/blurred vision	□ Yes □ No	□ less than 1 month □ 1-3 months □ over 3 to 6 months □ more than 6 months

Alternated smell	□ Yes □ No	□ less than 1 month □ 1-3 months □ over 3 to 6 months □ more than 6 months
Loss of smell	□ Yes □ No	□ less than 1 month □ 1-3 months □ over 3 to 6 months □ more than 6 months
Alternated taste	□ Yes □ No	□ less than 1 month □ 1-3 months □ over 3 to 6 months □ more than 6 months
Loss of taste	□ Yes □ No	□ less than 1 month □ 1-3 months □ over 3 to 6 months □ more than 6 months
Tremor/shakiness	□ Yes □ No	□ less than 1 month □ 1-3 months □ over 3 to 6 months □ more than 6 months
Tingling feeling/ “pins and needles“	□ Yes □ No	□ less than 1 month □ 1-3 months □ over 3 to 6 months □ more than 6 months
Seizures/fits	□ Yes □ No	□ less than 1 month □ 1-3 months □ over 3 to 6 months □ more than 6 months
Confusion/lack of concentration	□ Yes □ No	□ less than 1 month □ 1-3 months □ over 3 to 6 months □ more than 6 months
Problems speaking or communicating	□ Yes □ No	□ less than 1 month □ 1-3 months □ over 3 to 6 months □ more than 6 months
Insomnia *(hard to fall asleep, hard to stay asleep)*	□ Yes □ No	□ less than 1 month □ 1-3 months □ over 3 to 6 months □ more than 6 months
Hypersomnia *(excessive daytime sleepiness or prolonged nighttime sleep)*	□ Yes □ No	□ less than 1 month □ 1-3 months □ over 3 to 6 months □ more than 6 months
**Fatigue**	□ Yes □ No	□ less than 1 month □ 1-3 months □ over 3 to 6 months □ more than 6 months
**Gastrointestinal problems**	**Yes/No**	**<u>If yes</u>**, what is the duration of symptoms
Weight loss	□ Yes □ No	□ less than 1 month □ 1-3 months □ over 3 to 6 months □ more than 6 months
Problems swallowing or chewing	□ Yes □ No	□ less than 1 month □ 1-3 months □ over 3 to 6 months □ more than 6 months
Poor appetite	□ Yes □ No	□ less than 1 month □ 1-3 months □ over 3 to 6 months □ more than 6 months
Diarrhea	□ Yes □ No	□ less than 1 month □ 1-3 months □ over 3 to 6 months □ more than 6 months
Stomach/ abdominal pain	□ Yes □ No	□ less than 1 month □ 1-3 months □ over 3 to 6 months □ more than 6 months
Feeling nauseous	□ Yes □ No	□ less than 1 month □ 1-3 months □ over 3 to 6 months □ more than 6 months

Feeling sick/ vomiting	□ Yes □ No	□ less than 1 month □ 1-3 months □ over 3 to 6 months □ more than 6 months
Constipation	□ Yes □ No	□ less than 1 month □ 1-3 months □ over 3 to 6 months □ more than 6 months
**Dermatologic problems**	**Yes/No**	**<u>If yes</u>**, what is the duration of symptoms
Bilateral conjunctivitis	□ Yes □ No	□ less than 1 month □ 1-3 months □ over 3 to 6 months □ more than 6 months
If yes, □ purulent □ non-purulent		
Lumps or rashes (purple/pink) on toes	□ Yes □ No	□ less than 1 month □ 1-3 months □ over 3 to 6 months □ more than 6 months
Skin rash<br>*If yes, tick all body areas that apply*	□ Yes □ No	□ less than 1 month □ 1-3 months □ over 3 to 6 months □ more than 6 months
Face	□ Yes □ No	□ less than 1 month □ 1-3 months □ over 3 to 6 months □ more than 6 months
Trunk (stomach or back)	□ Yes □ No	□ less than 1 month □ 1-3 months □ over 3 to 6 months □ more than 6 months
Arms	□ Yes □ No	□ less than 1 month □ 1-3 months □ over 3 to 6 months □ more than 6 months
Legs	□ Yes □ No	□ less than 1 month □ 1-3 months □ over 3 to 6 months □ more than 6 months
Buttocks	□ Yes □ No	□ less than 1 month □ 1-3 months □ over 3 to 6 months □ more than 6 months
Toes	□ Yes □ No	□ less than 1 month □ 1-3 months □ over 3 to 6 months □ more than 6 months
Fingers	□ Yes □ No	□ less than 1 month □ 1-3 months □ over 3 to 6 months □ more than 6 months
Accompanied by itch	□ Yes □ No	□ less than 1 month □ 1-3 months □ over 3 to 6 months □ more than 6 months
**Cardiovascular problems**	**Yes/No**	**<u>If yes</u>**, what is the duration of symptoms
Palpitations (heart racing)	□ Yes □ No	□ less than 1 month □ 1-3 months □ over 3 to 6 months □ more than 6 months
Variations in heart rate (tachycardia or bradycardia)	□ Yes □ No	□ less than 1 month □ 1-3 months □ over 3 to 6 months □ more than 6 months
Bleeding	□ Yes □ No	□ less than 1 month □ 1-3 months □ over 3 to 6 months □ more than 6 months
If yes, specify bleeding site:		

**Genitourinary problems**	**Yes/No**	**If yes**, what is the duration of symptoms	**If yes**, what is the duration of symptoms
Urination problems	□ Yes □ No	□ less than 1 month □ 1-3 months □ over 3 to 6 months □ more than 6 months	
Changes in menstruation, if settled before Covid-19	□ Yes □ No □ Not applicable	□ less than 1 month □ 1-3 months □ over 3 to 6 months □ more than 6 months	

If your child had experienced/is experiencing any **other NEW symptoms** that were not covered by the tables above, please, specify them here. Please also indicate how long did these symptoms last (eg days, weeks, months or they are ongoing). **When did these symptoms start:**

PARTICIPANT IDENTIFICATION# : [__][_____][_____][_____][_____

We would like to know how good or bad your child's health was
BEFORE COVID-19 and is **TODAY**

This scale is numbered from 0 to 100%
with **100% meaning the best health** you can imagine
0% means the worst health you can imagine.

Please **write the number in the box below each scale** to indicate how good or bad your child's health was **BEFORE COVID-19** and is **TODAY**.

**Before COVID**	**Today**
100	100
95	95
90	90
85	85
80	80
75	75
70	70
65	65
60	60
55	55
50	50
45	45
40	40
35	35
30	30
25	25
20	20
15	15
10	10
5	5
0	0
☐	☐

Has your child been vaccinated in accordance with the national vaccination schedule?

☐ Yes, vaccinated up to date ☐ Yes, but some vaccines were missed ☐ No, I avoid vaccination for my child

PARTICIPANT IDENTIFICATION# : [__][_____][_____][_____][_____

Please provide an approximate date of your child's latest vaccination? [_D_][_D_]/[_M_][_M_]/[_2_][_0_][_Y_][_Y_]

Please, specify what was the vaccine: ___________________□ I do not remember

I trust information I receive about shots?
□ Not at all □ Only a little □ Quite a lot □ A great deal

How concerned are you that any of the childhood shots might not be safe?
□ Not at all □ Only a little □ Quite a lot □ A great deal

Has your child been vaccinated with vaccine against Covid-19?

□ Yes □ No □ Not sure

If no, would you like to vaccinate your child against Covid-19 in the future?

□ Yes □ No □ Not sure

I trust information I receive Covid-19 vaccination?
□ Not at all □ Only a little □ Quite a lot □ A great deal

How concerned are you that Covid-19 vaccination might not be safe?
□ Not at all □ Only a little □ Quite a lot □ A great deal

**Please let us know of any further comments about the child's illness, the pandemic, lockdown and/or any sequelae.**

End of survey

Thank you for your time!
